# Exome sequencing identifies a novel and a recurrent *BBS1* mutation in Pakistani families with Bardet-Biedl syndrome

**Published:** 2013-03-21

**Authors:** Muhammad Ajmal, Muhammad Imran Khan, Kornelia Neveling, Ali Tayyab, Sulman Jaffar, Ahmed Sadeque, Humaira Ayub, Nasir Mahmood Abbasi, Moeen Riaz, Shazia Micheal, Christian Gilissen, Syeda Hafiza Benish Ali, Maleeha Azam, Rob W. J. Collin, Frans P. M. Cremers, Raheel Qamar

**Affiliations:** 1 Department of Biosciences, Faculty of Science, COMSATS Institute of Information Technology, Islamabad, Pakistan; 2 Department of Human Genetics, Radboud University Nijmegen Medical Centre, Nijmegen, The Netherlands; 3 Shifa College of Medicine, Shifa Tameer-e-Millat University, Islamabad, Pakistan; 4 Institute for Genetic and Metabolic Disease, Radboud University Nijmegen Medical Centre, Nijmegen, The Netherlands; 5 Department of Ophthalmology, Shifa International Hospital, Islamabad, Pakistan; 6 Department of Ophthalmology, Radboud University Nijmegen Medical Centre, Nijmegen, The Netherlands; 7 Nijmegen Centre for Molecular Life Sciences, Radboud University Nijmegen Medical Centre, Nijmegen, The Netherlands; 8 Al-Nafees Medical College & Hospital, Isra University, Islamabad, Pakistan

## Abstract

**Purpose:**

To determine the genetic cause of Bardet-Biedl syndrome (BBS) in two consanguineous Pakistani families.

**Methods:**

Clinical characterization of the affected individuals in both families was performed with ophthalmic examination, electroretinography, electrocardiography, and liver and renal profiling. Seventeen genes are known to be associated with BBS, so exome sequencing was preferred over candidate gene sequencing. One affected individual from both families was selected for exome sequencing. Segregation of the identified variants was confirmed with Sanger sequencing.

**Results:**

Retinitis pigmentosa, obesity, and learning difficulties were present in the affected individuals in both families. In family A, a sixth finger (polydactyly) of the proband’s sister was removed by a surgical operation leaving a scar on the little finger. Polydactyly was also present in both affected individuals from family B. All diagnostic symptoms were characteristic of BBS in both families. In both affected individuals from family A, exome sequencing identified a novel homozygous mutation (c.47+1G>T) in *BBS1* that inactivates the splice donor site at the end of exon 1. In family B, a previously reported mutation, c.442G>A; p.(Asp148Asn), was detected.

**Conclusions:**

Exome sequencing is an efficient and cost-effective technique for identifying mutations in genetically heterogeneous diseases. In addition, intrafamilial phenotypic variability in family A argues for the modifying effect of other still unknown modifier alleles.

## Introduction

Bardet-Biedl syndrome (BBS; OMIM: 209900) is invariantly characterized by rod-cone dystrophy, and at least three additional non-ocular features such as intellectual disability, obesity, polydactyly, hypogonadism, or renal anomalies as primary manifestations. In the absence of one of these four primary clinical features, the diagnosis of BBS is made when at least two secondary features are observed, including hepatic fibrosis, diabetes mellitus, reproductive and developmental abnormalities, growth retardation, speech delays, or cardiovascular problems [1].

BBS has been classified as a ciliopathy [2] and is inherited mostly in an autosomal recessive pattern although digenic (triallelic) inheritance has also been reported [3-6]. Some studies have also demonstrated the epistatic effects of a third protective allele [7,8], while variants in *RPGRIP1L* have been reported as a modifier of the BBS phenotype [9]. In only two studies, a recessive mode of inheritance for BBS has been argued against [5,10]. Recently, a spectrum of phenotypes ranging from full-blown BBS to non-syndromic retinitis pigmentosa was found to be associated with the hypomorphic *BBS1* missense mutation p.Met390Arg [11].

BBS is a severe disorder with the highest prevalence of 1/3,700 in the Faroe Islands [12]. Although in the rest of the world the prevalence of BBS varies from 1/13,000 in Newfoundland [13] to 1/17,000 in the Kuwaiti population [14] and 1/65,000 in other Arab countries [15], BBS is rare in the European population with a prevalence range of 1/125,000 in the UK [16] to 1/160,000 in Switzerland [17].

BBS is genetically heterogeneous, as mutations in 17 different genes have been identified so far [18]. The *BBS1* gene (MIM: 209901) is located on the long arm of chromosome 11 and consists of 17 coding exons. The gene is expressed in many tissues, including fetal, testicular, retinal, adipose, cardiac, skeletal, and pancreatic cells, with the highest expression in the kidney [19]. BBS1 is part of the BBSome complex that includes BBS1, BBS2, BBS4, BBS5, BBS7, BBS8, and BBS9. Proteins in this complex are thought to be involved in ciliogenesis, because of their function in membrane trafficking in the primary cilium [20].

The current study was designed to find the molecular basis of BBS in two Pakistani families (families A and B) using exome sequencing. A novel splice donor site mutation c.47+1G>T in *BBS1* was identified in family A, and a previously reported *BBS1* mutation, c.442G>A; p.(Asp148Asn) [21], was identified in family B.

## Methods

### Ethics committee/institutional review board approval

Approval for this study was granted by the ethics committee/institutional review board of Shifa College of Medicine, Shifa International Hospital, Islamabad. Written informed consent was obtained from both families before the study began. In addition, the study conformed to the tenets of the Declaration of Helsinki.

### Clinical examination

Clinical examination included ophthalmic examination, electroretinography, echocardiography, liver function tests, and renal profiling. Blood glucose was also measured to determine the presence or absence of diabetes mellitus.

### Blood sampling and DNA preparation

**Figure 1:**
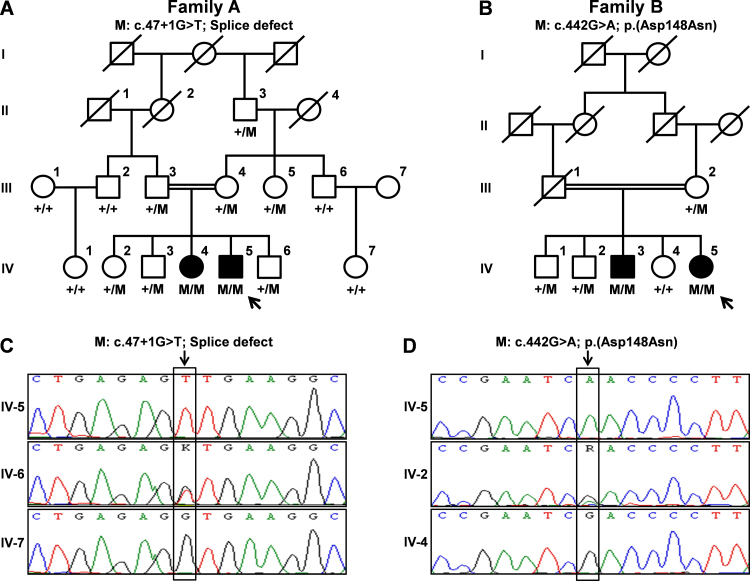
Pedigrees of families A and B and sequence electropherograms. **A**: Segregation of the identified mutation in family A. **B**: Segregation of the identified mutation in family B. **C**: Sequence electropherograms of an affected individual (upper panel), a heterozygous mutation carrier (middle panel), and a homozygous wild-type healthy individual (lower panel) of family A. **D**: Sequence electropherograms of an affected person (upper panel), a heterozygous mutation carrier (middle panel), and a homozygous wild-type healthy individual (lower panel) of family B. In family pedigrees, roman numerals indicate generation number, arrows indicate probands, M stands for mutation identified, + is wild-type allele, M/M indicates genotypes of affected individuals, +/M indicates genotypes of unaffected individuals carrying a mutant allele, and +/+ indicates genotypes of healthy individuals.

Both families (Figure 1A,B) were recruited from the central part of Punjab. Venous blood of affected and normal individuals of both families was drawn by venipuncture and collected in acid citrate dextrose vacutainers (Becton Dickinson, Franklin Lakes, NJ). DNA was extracted with a standard phenol-chloroform extraction procedure [22]. Briefly, it consisted of the lysis of white blood cells, followed by protein digestion, extraction of the DNA with phenol-chloroform, and precipitation of DNA with isopropanol.

### Exome sequencing

Probands of each family were selected for exome sequencing, which was performed on a 5500XL sequencing platform from Life Technologies (Carlsbad, CA). The exomes of the probands were enriched according to the manufacturer’s protocol using the SureSelect Human All Exon v2 Kit (50 Mb), containing the exonic sequences of approximately 21,000 genes from Agilent Technologies, Inc. (Santa Clara, CA). LifeScope software v2.1 from Life Technologies (Carlsbad, CA) was used to map color space reads along the hg19 reference genome assembly. The DiBayes algorithm, with high-stringency calling, was used for single-nucleotide variant calling. The small Indel Tool was used to detect small insertions and deletions. Exome sequencing data were filtered as described previously [23,24]. Briefly, the variants in known BBS genes were selected and analyzed for segregation in the families.

### Sequence analysis

Variants identified with exome sequencing were confirmed with Sanger sequencing using an automated DNA sequencing machine (3730 DNA analyzer; Applied Biosystems, Inc., Foster City, CA). PCR primers were designed with the help of the online tool Primer3 [25].

### Splice site prediction

NetGene2 World Wide Web Server [26,27], an online splice site prediction program, was used to predict the effect of sequence variants located in or near the splice sites.

### In silico pathogenicity assessment of missense variant

Pathogenicity of the missense variant was assessed using the online prediction tools polymorphism phenotyping v-2 (PolyPhen-2) and sorting intolerant from tolerant (SIFT). In addition, the HOPE server was used to predict structural consequences in the mutant protein using normal protein structure with accession number Q8NFJ9 [28].

## Results

### Clinical findings

**Figure 2:**
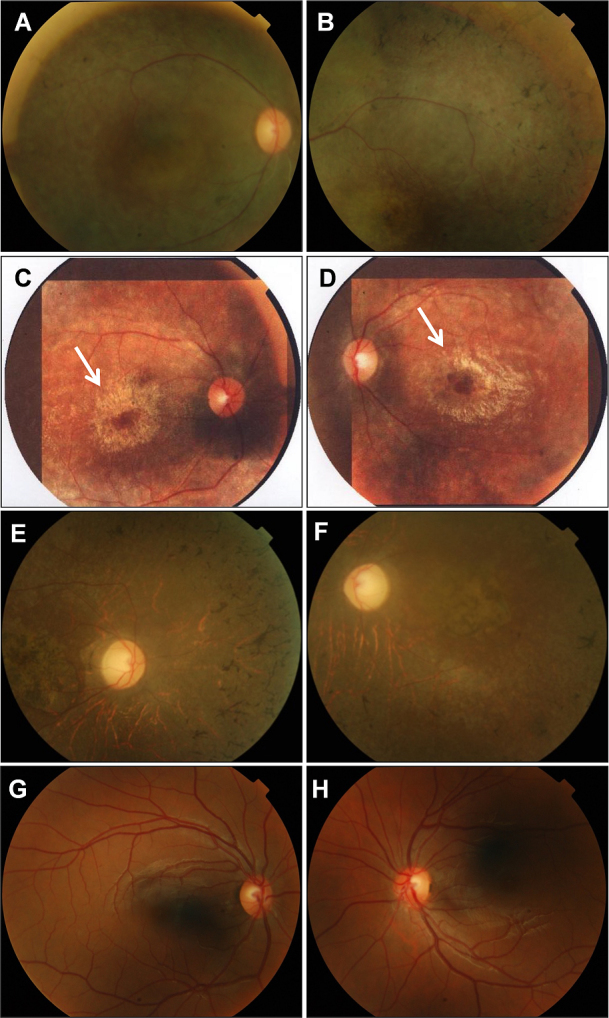
Fundus photographs of affected and healthy individuals. **A**, **B**: Fundus photographs of family A, proband IV:5, show bone spicules, retinal vessel attenuation, and macular degeneration. **C**, **D**: Fundus photographs of the proband’s sister (IV:4) show the salt-and-pepper appearance of both fundi, the presence of bone spicules in the midperiphery, and bull’s eye macular atrophy (indicated by the arrows). **E**, **F**: Fundus photograph of family B proband IV:5 reveals pigmentary deposits and retinal vessel attenuation. **G**, **H**: Fundus photographs of a healthy individual from family A (IV:6).

Affected individuals underwent extensive clinical examination, including fundus examination, which revealed the presence of bone spicules and attenuation of blood vessels (Figure 2). Electroretinography measurements were recorded for affected individual IV:5 of family A only, which showed reduced scotopic and photopic electrophysiological responses in the patient compared to a normal individual (Table 1). All other primary and secondary BBS features were also investigated (Table 2). Retinitis pigmentosa, obesity, and learning difficulties were present in all affected individuals while polydactyly was present in the affected woman (IV:4) of family A and affected individuals IV:3 and IV:5 of family B. Developmental delay, a secondary feature of BBS, was also observed in both families. Echocardiography was normal in both affected individuals of family A whereas in family B echocardiography was not performed, but affected individuals of family B were hypertensive. Liver function was also normal, and no renal defects were observed in either family. Affected individuals of family B also had intellectual disability and dental crowding. All patients fulfilled the diagnostic criteria of BBS having at least four primary or three primary and two secondary BBS features.

**Table 1:** Electrophysiological measurements recorded for individual IV:5 of family A.

**Measured parameters using monopolar electrodes**	**Adaptation**	**Flash strength** **(cd.s/m^2^)**	Proband*	**Control**	**Standard Values** **(Age=30 years)**
Scotopic 25 dB b-wave amplitude (µV)	Dark	0.01	9.5	177.5	>163
Scotopic 0 dB b-wave amplitude (µV)	Dark	3.0	7.1	434.4	>403
Oscillatory potential amplitude (µV)	Dark	3.0	30.2	193.1	>89
Photopic 0 dB b-wave amplitude (µV)	Light	3.0	7.6	123.6	>92
Photopic 30 Hz flicker amplitude (µV)	Light	3.0	3.2	53.3	>63

*Proband’s age was 26 at the time of the measurements.

**Table 2:** BBS features in affected individuals of both families.

**BBS features**	**Present**		
**Primary features**	Family A IV:5	Family A IV:4	Family B IV:5
1. Rod-cone dystrophy	Yes	Yes	Yes
2. Polydactyly	No	Yes	Yes
3. Obesity	Yes	Yes	Yes
4. Learning problems	Yes	Yes	Yes
5. Hypogonadism	No	No	No
6. Renal malfunction	No	No	No
**Secondary features**			
1. Speech problems	No	No	Yes
2. Strabismus, cataract, astigmatism	Strabismus	Astigmatism	No
3. Brachydactyly, syndactyly	No	No	No
4. Developmental delay	Yes	Yes	No
5. Polyuria, polydipsia	No	No	No
6. Diabetes mellitus	No	No	No
7. Ataxia, imbalance	No	No	No
8. Mild spasticity	No	No	No
9. Dental crowding	No	No	Yes
10. Heart problems	No	No	Yes
11. Liver disease	No	No	No
12. Family members with BBS	Yes	Yes	Yes

### Genetic findings

To identify the pathogenic mutation, the exome data sequence variants were filtered to reduce the number of potentially pathogenic variants. First, we searched for variants present in known BBS genes. In both families, variants were found in six BBS genes, including *BBS1*, *BBS2*, *BBS4*, *BBS7*, *BBS9*, and *BBS12* (Table 3). The frequency of the variants identified in known BBS genes, except the *BBS1* variants described below, ranged from 13% to 99% in an in-house database.

**Table 3:** Exome sequencing variants in previously implicated BBS genes

**Family A**										
**Chr**	**Reads**	**Var reads**	**% var**	**SNP id**	**Freq**	**Gene**	**AA changes**	**mRNA changes**	**phyloP**	**GS**
11	2	2	100	-	-	*BBS1*	-	c.47+1G>T	3.03	-
16	109	106	97	rs4784677	97.09	*BBS2*	p.(Ser70Asn)	c.209C>T	2.50	46
16	119	46	39	rs11373	28.48	*BBS2*	p.(Ile123Val)	c.367T>C	0.23	29
15	45	44	98	rs8033604	76.70	*BBS4*	-	c.77–6 G>A	−3.73	-
15	103	103	100	rs12914333	99.35	*BBS4*	p.(Phe302Phe)	c.906T>C	−0.58	-
15	20	20	100	rs2277598	78.64	*BBS4*	p.(Ile354Thr)	c.1061T>C	−0.39	89
4	68	22	32	rs1507994	12.94	*BBS7*	-	c.1890+16G>A	−0.92	-
7	87	27	31	rs11773504	35.92	*BBS9*	p.(Ala455Thr)	c.1363G>A	0.31	58
4	41	23	56	rs309370	51.46	*BBS12*	p.(Arg386Gln)	c.1157G>A	−0.34	43
4	99	62	63	rs13102440	36.57	*BBS12*	p.(Gln624Gln)	c.1872A>G	−0.05	-

**Family B**										
**Chr**	**Reads**	**Var reads**	**% var**	**SNP id**	**Freq**	**Gene**	**AA changes**	**mRNA changes**	**phyloP**	**GS**
chr11	51	51	100	-	-	*BBS1*	p.(Asp148Asn)	c.442G>A	3.54	23
chr16	92	92	100	rs4784677	97.02	*BBS2*	p.(Ser70Asn)	c.209C>T	2.50	46
chr15	73	72	99	rs12914333	99.19	*BBS4*	p.(Phe302Phe)	c.906T>C	−0.51	-
chr15	31	13	42	rs2277598	76.42	*BBS4*	p.(Ile354Thr)	c.1061T>C	−0.32	89
chr4	49	47	96	rs309370	52.85	*BBS12*	p.(Arg386Gln)	c.1157G>A	−0.32	43
chr4	50	15	30	rs13135766	29.27	*BBS12*	p.(Val460Val)	c.1380G>C	1.66	-
chr4	62	28	45	rs13135445	34.96	*BBS12*	p.(Cys470Cys)	c.1410C>T	−0.39	-
chr4	84	36	43	rs13102440	36.86	*BBS12*	p.(Gln624Gln)	c.1872A>G	0.01	-

AA, amino acid; Chr, chromosome; Freq, frequency; GS, Grantham score; phyloP, phylogenetic p values; SNP id, single nucleotide polymorphism identification; Var, variation

**Figure 3:**
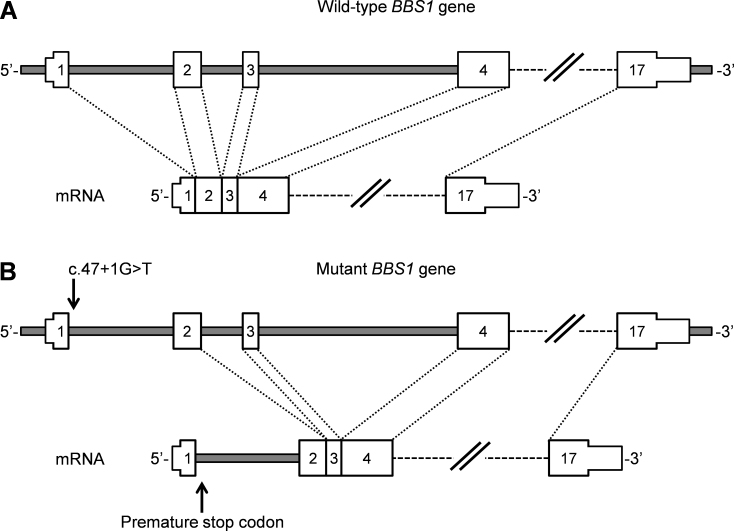
Predicted effect of splice donor site mutation c.47+1G>T on mutant *BBS1* messenger RNA splicing. **A**: Wild-type *BBS1* and resulting messenger RNA (mRNA) after splicing are shown. **B**: Mutant *BBS1* and inclusion of intron 1 in the mutant mRNA are indicated, which might result in nonsense-mediated decay of the RNA or the synthesis of a truncated BBS1 protein.

In family A, a novel splice donor site mutation (c.47+1G>T) in *BBS1* was identified (Table 3). This mutation was present homozygously in the exome data with only two reads, which was further confirmed as a homozygous change with Sanger sequencing (Figure 1C). Segregation analysis (Figure 1A) revealed that the mutation was homozygous in both affected siblings, heterozygous in the parents and normal siblings, and absent in other unaffected members of the family. The c.47+1G>T variant is predicted to affect one of the canonical splice site nucleotides, which might therefore completely inactivate splice donor site. Splice site prediction software predicted inactivation of the wild-type splice donor site at the 3′ end of exon 1 and did not predict an alternative splice donor site in intron 1. The mutant messenger RNA is likely to have a premature stop codon at position 17 (Figure 3).

Similarly in family B, exome data were analyzed for variants in known BBS genes that resulted in identifying a previously reported missense mutation c.442G>A; p.Asp148Asn in *BBS1* (Figure 1D; Table 3). Segregation analysis (Figure 1B) revealed that this mutation was homozygously present in the affected individuals (IV:3 and IV:5), heterozygously carried by the healthy siblings (IV:1 and IV:2) and their mother (III:2) and absent in one healthy sister (IV:4).

Polyphen-2 predicted missense mutation p.(Asp148Asn) was “probably damaging” whereas SIFT predicted this mutation was “tolerated.” HOPE predicted that the mutation is present in the core of a domain; a difference in the properties of the wild-type and mutant amino acid residues might disturb the core structure of this domain.

## Discussion

In the current study, we report on two consanguineous families from Pakistan, with affected individuals presenting BBS, a rare, clinically and genetically heterogeneous disorder. In approximately 75% of families with BBS, mutations are detected in known BBS genes [21]. *BBS1* is the most frequently mutated BBS gene and accounts for the disease in nearly 24% of European patients with BBS [4,29,30]. The *BBS1* mutation p.(Met390Arg) is a frequent founder mutation [5,7,19] found in 78.3% of families with *BBS1* mutations [5,21].

Including our families, more than 25 different Pakistani families have been described with variable BBS phenotypes and different mutations in *BBS2*, *BBS3*, *BBS4*, *BBS5*, *BBS8*, *BBS10*, and *BBS12* [31-36]. *BBS1* mutations have not been reported previously in Pakistani individuals with BBS.

The exact function of BBS1 in the pathology of BBS is still unclear but as a part of the BBSome complex, BBS1 is thought to play a crucial role by interacting with other proteins through its beta-propeller domain [37]. A detailed model of the assembly of the BBSome complex was proposed recently [38], which describes crucial steps required for the proper assembly of BBS proteins to form a functional BBSome complex. BBS1 joins the BBSome complex by interacting with BBS9 and BBS2, and the last component (BBS4) completes the BBSome complex assembly [38]. In the presence of the BBS1^M390R/M390R^ mutant protein, BBS4 fails to join the BBSome complex, which shows that protein–protein interactions between mutant BBS1 and wild-type BBS4 are lost [38]. The p.(Met390Arg) variant is a frequently occurring missense mutation in *BBS1* in individuals with BBS that severely affects these normal protein–protein interactions.

The splice site mutation c.47+1G>T identified in the current study abolishes the splice site and theoretically could result in the synthesis of a truncated mutant protein as a consequence of intron inclusion. Truncation most likely, however, results in nonsense-mediated decay of the messenger RNA because of the possible creation of a premature stop codon at position 17 (Figure 3); alternatively, use of an alternate downstream translation initiation codon ATG might result in 5′ truncation causing synthesis of a misfolded/non-functional protein. Misfolded proteins are mostly likely triggered toward the degradation pathways, which safeguards the cells from toxicity of the accumulating intermediary molecules [39-41].

In family A, the main intrafamilial phenotypic difference was the presence of polydactyly. In addition, bull’s eye macular atrophy was observed only in the affected woman, which also highlights the phenotype variability. Polydactyly is a common clinical feature of BBS [18]; the absence of polydactyly in the proband of family A could be the effect of a still unknown modifier allele. A known modifier of ciliopathies (*RPGRIP1L*) has been functionally tested in zebrafish to observe the modifying effects of different alleles [9]. In our family, variants were not identified in *RPGRIP1L*. Moreover, although we did not test the functional effect of other identified variants on phenotype, their effect on phenotypic variability cannot be ruled out. Ophthalmological examinations revealed severely reduced electrophysiological responses of rods and cones (Table 1), and the visual acuity was restricted to hand movements only, illustrating the severity of the disease. The proband of family B had intellectual disability and did not cooperate during the electrophysiological measurements.

Contrary to our findings, in a recent study, *BBS1* mutations were reported to be associated with milder ocular phenotypes compared to phenotypes associated with mutations in other BBS genes [42]. Moreover, in another study, milder non-ocular BBS phenotypes were reported in patients with mutations in *BBS12* [35]. In addition, mutations in *BBS1* [11], *BBS3* [43], and *BBS8* [44] have also been implicated in non-syndromic retinitis pigmentosa.

In a pharmacogenomic study aberrant splicing caused by a splice donor site mutation in *BBS1* (c.479G>A) was corrected in the patient’s fibroblasts by using mutated U1 small nuclear RNA (snRNA) [45]. U1 snRNA is involved in the recognition of exons during splicing. The investigators generated mutant U1 snRNAs with increased complementarity for a mutated splice donor site, which were subsequently used to redirect correct splicing. Using a similar strategy, the splice donor site mutation (c.47+1G>T) identified in our study could first be assessed in in vitro studies and then in model organisms, which might then lead to the development of treatment options for individuals with this particular mutation in the future.

The missense mutation p.(Asp148Asn) was previously identified in an American and a British patient [21]; thus, this is the second report of this mutation. Although this mutation is distributed worldwide, it is rare, being a genetic cause of the disease in only four patients.

In the absence of a solved three-dimensional structure or modeling template for the wild-type BBS1 protein, the program HOPE did not predict a three-dimensional model for the mutant protein, but based on the differences in the amino acid properties, it was predicted that wild-type interactions of the protein might be disturbed due to the introduction of a mutant residue. Although in silico analysis is a good analytical tool for assessing the pathogenicity of missense variants, functional validation is mandatory.

The use of exome sequencing to identify genetic mutations in BBS families was justified in our study when we compared the expenses of Sanger sequencing and exome sequencing. Expenditure for the Sanger sequencing (€2,140) of the coding exons (228 exons; 214 amplicons) of 17 BBS genes in both directions is comparable with the exome sequencing costs (€1,500–2,000). In addition, Sanger sequencing requires longer hands-on time and more effort compared to exome sequencing.

In conclusion, in Pakistani families with BBS, exome sequencing proved to be a successful and fast method for identifying a novel mutation and a recurrent mutation in *BBS1*. To our knowledge, this is the first report describing *BBS1* mutations in the Pakistani population. In the future, some of the clinical features might be addressed using gene therapy, but currently, only genetic counseling is warranted for carriers of mutations.
